# The quantitative assessment of microvascular obstruction size using first-pass perfusion cardiac MR

**DOI:** 10.1186/1532-429X-13-S1-P136

**Published:** 2011-02-02

**Authors:** Yoko Mikami, Andreas Kumar, Vanessa M Ferreira, Mouhieddin Traboulsi, Todd J Anderson, Matthias G Friedrich

**Affiliations:** 1 Stephenson Cardiovascular MR Centre, Libin Cardiovascular Institute of Alberta, Calgary, AB, Canada; 2 Laval University, The Institute of Cardiology and Pulmonology of Quebec, Quebec, QC, Canada; 3 University of Oxford Centre for Clinical Magnetic Resonance Research, Oxford, UK; 4 University of Calgary, Calgary, AB, Canada; 5 University of Calgary, Libin Cardiovascular Institute of Alberta, Calgary, AB, Canada

## Background

The presence and absolute amount of microvascular obstruction (MO) are associated with adverse left ventricular remodelling and prognosis in patients with acute myocardial infarction. MO can be detected with cardiovascular magnetic resonance imaging (CMR) using the technique of first pass perfusion imaging, late contrast enhanced CMR or early contrast enhanced CMR. It has not been shown which standardized definition of hypoenhancement related to the signal of remote myocardium is appropriate to evaluate the presence and the extent of MO on first pass perfusion images.

## Methods

We studied 52 patients with reperfused acute myocardial infarction which showed MO defined as persistent subendocardial hypoenhancement on rest perfusion images. We analyzed rest first-pass perfusion images, early contrast enhanced images and late contrast enhanced images scanned in a 1.5-T magnetic resonance imaging system. The size of MO was measured on rest perfusion images visually and quantitatively using signal intensity (SI) below 2SD and 3SD of remote myocardium as a threshold. The size of MO was also measured visually on early contrast enhanced images and quantitatively on late contrast enhanced images quantitatively defined as the area which did not show SI more than 5 SD above the remote myocardium surrounded by abnormal enhancement.

## Results

On rest perfusion images, standardized analysis using the threshold of −2SD of remote myocardium showed good agreement with visual analysis (ICC= 0.793). Mean MO size using threshold of −2SD of remote myocardium was not significantly different from those by visual assessment (2.22±1.25 cm^2^ and 2.40±1.25 cm^2^, p=N.S.), whereas those using threshold of −3SD of remote myocardium was significantly smaller (1.00±0.85 cm^2^, p<0.05). MO size analyzed by early contrast enhanced images showed good agreement with visual analysis on perfusion images (ICC=0.881). Mean MO size assessed by late contrast enhanced images was significantly smaller than those on by visual analysis on perfusion images (1.16±1.21 cm^2^ and 2.40±1.27 cm^2^, p<0.005).

## Conclusions

In patients with acute, reperfused MI, standard late enhancement images underestimate the size of myocardial areas with microvascular obstruction. Using a cutoff of 2 SD below mean SI of remote myocardium, rest perfusion images should be used to quantify the extent of microvascular obstruction.

